# A nucleic acid labeling chemistry reveals surface DNA on exosomes

**DOI:** 10.1101/2025.11.18.689134

**Published:** 2025-11-19

**Authors:** Filip Boskovic, Priyanka Dutta Gupta, Jian Zhang, Yamuna Krishnan, Jack W. Szostak

**Affiliations:** 1 Howard Hughes Medical Institute, University of Chicago, Chicago, IL, 60637, USA; 2 Department of Chemistry, University of Chicago, Chicago, IL, 60637, USA; 3 Neuroscience Institute, The University of Chicago, Chicago, IL, 60637, USA; 4 Institute for Biophysical Dynamics, The University of Chicago, Chicago, IL, 60637, USA; 5 These authors contributed equally.

**Keywords:** nucleic acid chemical labeling, nucleic acid chemistry, surface DNA, exosomes, Major: Physical Sciences, Minor: Chemistry, Major: Biological Sciences, Minor: Immunology and Inflammation

## Abstract

Chemical labeling of nucleic acids is essential to pinpoint the structure, localization, and function of RNA and DNA. Yet, reversible sequence-independent chemistries that can label native RNA and DNA remain poorly developed. Here we describe Reversible Uridine Nitrilium-mediated Addition (RUNA), a reversible covalent chemistry that selectively modifies uridine and thymidine residues *via* N3 deprotonation and reaction with a nitrilium ion intermediate generated from an aldehyde and an isonitrile. The reaction forms a stable N3 adduct that can be quantitatively reversed by hydrolysis. By labeling uridines and thymidines sequence-independently with reagents that are either membrane permeable or impermeable, we pinpointed the localization and function of DNA on exosomes. Although exosomes harbor nucleic acids, whether the latter are encapsulated in the exosome lumen or are surface-adhered is unknown. RUNA revealed that exosomal DNA is surface-exposed. The abundance of such surface-bound DNA increases upon DNA-damage accumulation in cancer cells that are treated with a PARP inhibitor. This surface-bound DNA drives exosome uptake by M2-polarized macrophages through scavenger receptors and triggers a shift toward an M1-like pro-inflammatory state. The selective labeling of surface DNA revealed an unexpected mechanism by which exosomes engage innate immune cells. RUNA is a versatile tool to analyze the nucleic acid content and functionality of extracellular vesicles in health and disease.

## Introduction

The introduction of a label on RNA or DNA, independent of sequence, requires the functionalization of either the nucleobase or the sugar-phosphate backbone (1–4). Such labels can include fluorophores for imaging, affinity handles for enrichment and purification, or reactive groups for bioorthogonal conjugation (5–9). Such tags enable the study of nucleic acid composition, structure, localization, and function in biology (10–14). While this is best achieved through enzymatic or chemical labeling strategies that are minimally disruptive, enzymatic methods generally require defined sequence contexts (15–17). Hence, sequence-independent labeling chemistries are highly desirable, as they allow unbiased analysis of nucleic acids. For example, RNA labeling by 2′-hydroxyl acylation or sulfonylation has led to the mapping of RNA structures and RNA-RNA interactions (1, 18). Nucleobase-specific chemistries using glyoxal and kethoxal that selectively label unpaired guanosines (19, 20) underpin techniques such as KAS-seq or KARR-seq, that map transcriptionally-active DNA loci (21) or RNA-RNA interactions, respectively (2). Carbodiimide reagents such as 1-ethyl-3-(3-dimethylaminopropyl)carbodiimide (EDC) and N-cyclohexyl-N′-(2-morpholinoethyl)carbodiimide (CMC), which modify uridine and guanosine, form the basis of pseudouridine sequencing (4, 22). Modular chemistries enable diverse applications because they are adaptable to an array of functional groups. Reversible chemistries would enable labeling that is sequence-independent yet temporally controllable.

New biological roles are emerging for membrane-associated nucleic acids in cellular communication and immune regulation (23–26). The importance of surface RNA highlights the need for mild, sequence-independent labeling strategies that can help to define their location, synthesis and functionality (26–28). While glycoRNAs have been shown to engage Siglec receptors (29) and modulate immune signaling, it is clear that much more remains to be elucidated (23–25). Circulating tumor DNA has become very important in cancer diagnosis (28, 30). Both tumor cells and normal cells release small extracellular vesicles, including exosomes, that have associated nucleic acids (23, 27, 30). However, it is unclear whether the DNA is housed inside the exosome or is surface associated (27). Although this distinction is important for understanding how exosomes mediate immune responses, it has remained unresolved because of the lack of chemistry to selectively and reversibly label membrane-associated nucleic acids.

To probe nucleic acids in such cell-derived compartments sequence-independently, we developed a reversible labeling chemistry that selectively modifies uridines and thymidines in RNA and DNA respectively. It couples an aldehyde and an isonitrile to generate a nitrilium ion *in situ*, which reacts specifically at the N3 position of uracil and thymine to form a stable covalent adduct. This reaction, termed Reversible Uridine Nitrilium-mediated Addition (RUNA), is highly modular. By changing the aldehyde one can install diverse functional groups on the DNA or RNA. If the tag is membrane-impermeant, one can probe nucleic acid accessibility across biological membranes.

We applied RUNA to resolve the long-standing conundrum of whether exosomal DNA is entrapped within the lumen or adhered to the outer surface (27, 30–32). By applying RUNA to exosomes derived from prostate cancer cells, we found that DNA is present on the surface of exosomes. When the prostate cancer cells are treated with a poly(ADP-ribose) polymerase (PARP) inhibitor such as rucaparib, the abundance of surface-attached DNA increased, revealing that DNA damage can influence the availability of extracellular DNA. Importantly, such exosomes are more efficiently internalized by M2-polarized macrophages, which then adopt a more M1-like macrophage state and a proinflammatory cytokine response. These results reveal exosomal DNA as both a determinant of exosome uptake and a modulator of macrophage phenotype. Our studies position RUNA as a versatile chemical platform for the reversible labeling of nucleic acids with spatial resolution across membranes. Our approach opens new avenues to explore the composition and function of membrane-associated RNA and DNA in physiology and disease.

## Results

### Mechanism of native RNA and DNA labeling with RUNA chemistry

**Figure 1.**
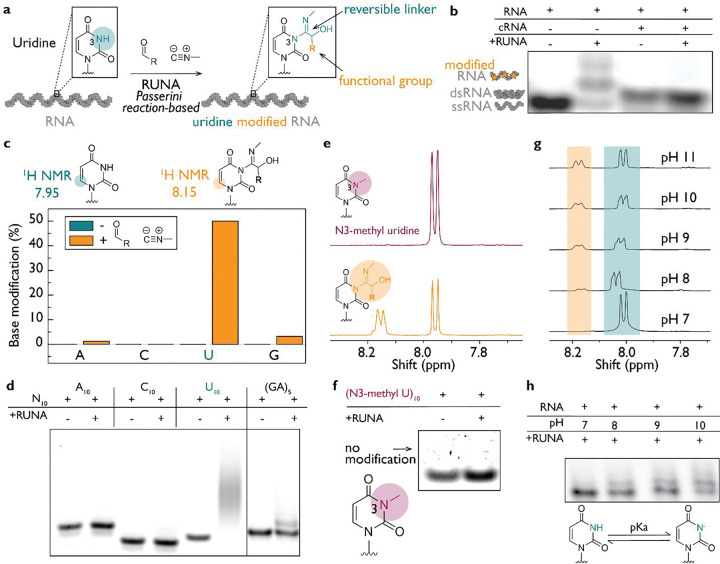
Nucleotide-specific RNA labeling involves N3 of uridine. (**a**) Uridine in RNA is modified to form an N3 adduct containing a removable linker and a functional group of interest. (**b**) Gel analysis shows that single-stranded RNA (12-nt Cy3-labeled) reacts with RUNA chemistry, while double-stranded RNA made by mixing RNA and complementary RNA (cRNA) is not modified. (**c**) The extent of N3 adduct formation is quantified using the diagnostic proton shift observed in the ^1^H NMR spectrum. Uridine (U) demonstrates a significantly higher modification rate compared to other canonical nucleotides. (**d**) Using 10-mer oligonucleotides with a 3′ Cy3 label, we showed that U_10_ undergoes modification, whereas other oligonucleotides exhibit limited reactivity. (**e**) The proposed modification site was validated by comparing N3-methyluridine (purple) with uridine (orange), showing that under identical conditions, modification occurs exclusively with uridine. (**f**) Using an N3-methyluridine oligonucleotide labeled with 3′ Cy3, we confirmed that no modification occurs in the absence of an accessible N3 position. (**g**) Further confirmation that the deprotonated N3 position undergoes modification was achieved by performing the reaction under varying pH conditions. The N3 pKa is reported to be approximately 9.2; we observed low modification at pH 7, whereas higher pH increased the extent of uridine modification. (**h**) Gel analysis performed at different pH levels corroborated the observations in (g), confirming that the reaction can be effectively controlled by pH. For all panels, the aldehyde 4-pentenal and methyl isonitrile were used.

**Scheme 1.**
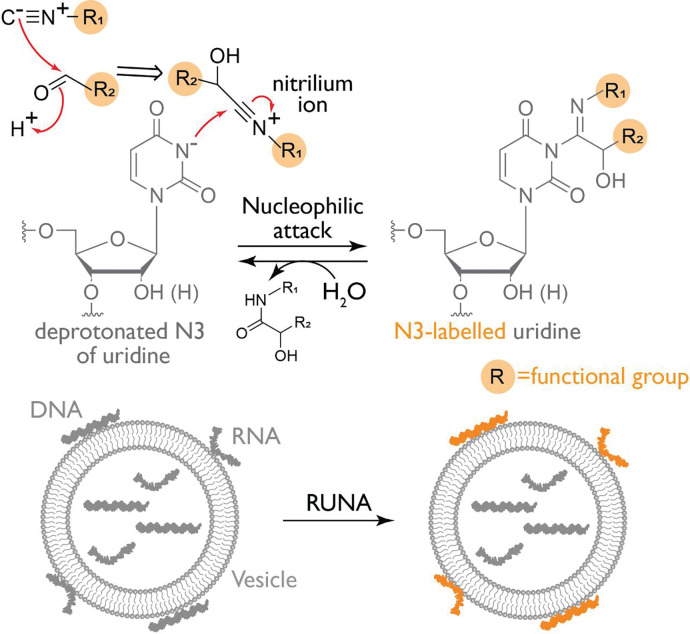
The proposed mechanism for the uridine-preferred labeling of N3 *via* an *in situ* formed nitrilium ion. The deprotonated N3 of uridine (or thymidine) acts as a nucleophile, attacking the nitrilium ion generated from the reaction of an aldehyde with an isonitrile (where R represents a functional group of interest). The resulting modification is hydrolyzed on demand by heating, thereby restoring the native RNA/DNA. The choice of functional groups enables membrane-specific labeling, allowing selective modification of extravesicular or surface-exposed nucleic acids.

To label nucleic acids reversibly and sequence-independently, we developed RUNA, a uridine- and thymidine-selective chemistry that proceeds *via* the *in situ* generation of a nitrilium ion intermediate. The reaction begins with the condensation of an aldehyde and an isonitrile to form a nitrilium intermediate, which is then attacked by the deprotonated N3 of uridine or thymidine, yielding a covalent adduct (33, 34). Because this modification is thermally labile it can be hydrolyzed by mild heating to restore the native nucleobase (Scheme 1). It can thus tunably and reversibly tag both RNA and DNA with multiple functional groups such as alkynes, alkenes, and strained cycloalkenes (Figure 1a).

We first sought to determine whether base pairing of uridine affects its reactivity to RUNA, employing methyl isonitrile (MeNC) and 4-pentenal. We compared the labeling efficiency of single-stranded RNA versus when it was part of an RNA duplex (Figure 1b). Using denaturing polyacrylamide gel electrophoresis (PAGE), we observed that single-stranded RNA was modified (lane 2), whereas duplex RNA exhibited limited uridine labeling (lane 4). Interestingly, RUNA labels both single-stranded and duplex DNA with high efficiency (Figure S1).

To determine nucleobase selectivity, we monitored RUNA labeling on the four canonical ribonucleotides using ^1^H nuclear magnetic resonance (NMR) spectroscopy. Only UMP showed a new diagnostic proton signal upon modification, consistent with the formation of an N3 adduct (Figure 1c; Figures S2–S9). When we rigorously tested this preference using short oligonucleotides, we found high selectivity for rU_10_, over rA_10_, rC_10_, and r(GA)_5_ (Figure 1d; Table S1). Further, U_10_ exhibits at least one modification per molecule unlike other oligonucleotides, where labeling was negligible under the same conditions. We observed similar reactivity for deoxyribonucleotides, showing that RUNA selectively targets U and T in RNA and DNA respectively (Figure S10).

We hypothesized that RUNA labels the N3 of uridine by deprotonation, followed by nucleophilic attack on the *in situ* generated nitrilium ion to yield the N3 adduct. To test this mechanism, we evaluated both free N3-methyl uridine (N3-Me U) and an N3-Me U oligomer with RUNA, and in both cases found that N3 methylation blocks uridine modification (Figure 1e–f, Figure S11). To rigorously test the need for N3 deprotonation, we examined the pH dependence of RUNA labeling (Figure 1g, h). Higher pH led to higher yields of the N3 adduct, consistent with its pKa of 9.2 (Figure S12). We tested a range of aldehydes at pH values from 7 to 10 and found that labeling was generalizable across aldehydes (Figure S13–S16). Although many are compatible with RUNA, long-chain aliphatic and highly hydrophobic aldehydes such as C8, C10, and pyrene derivatives do not support efficient modification, primarily due to solubility problems (Figure S20).

We next evaluated potential side reactions and delineated the scope of isonitriles and aldehydes compatible with RUNA. One possible side reaction involves formation of a 5′-imidoyl phosphate intermediate, which could undergo a Passerini-type rearrangement to yield a stable phosphodiester byproduct (33). However, this byproduct was undetectable under our conditions (Figure S17). The isonitrile moiety must be attached to a primary carbon for efficient nitrilium ion formation, since secondary or tertiary substitutions led to poor yields (Figure S18). As an alternative to the volatile methyl isonitrile, we found that 2-morpholinoethylisonitrile supported efficient labeling (Figure S19). Indeed, this chemistry modifies RNA and DNA with various reactive groups such as alkenes, alkynes, and strained cycloalkenes (Figures S13–S16).

Lastly, we found that RUNA can label RNA with norbornene aldehyde, which is amenable to click chemistry, with an observed rate of 0.33 ± 0.01 min^−1^ (Figure S21). The resulting norbornene-modified RNA reacted with biotin-PEG_4_-tetrazine through an inverse electron-demand Diels–Alder reaction (IEDDA), thereby tagging the RNA at a rate of 23.4 ± 4.8 min^−1^ (Figure S22). In sum, RUNA labeling was remarkably efficient in near-native environments of physiological ion levels, pH and temperature, molecular crowding and low mM reagent concentrations (Figure S23).

### Reversible functionalization of native RNA

**Figure 2.**
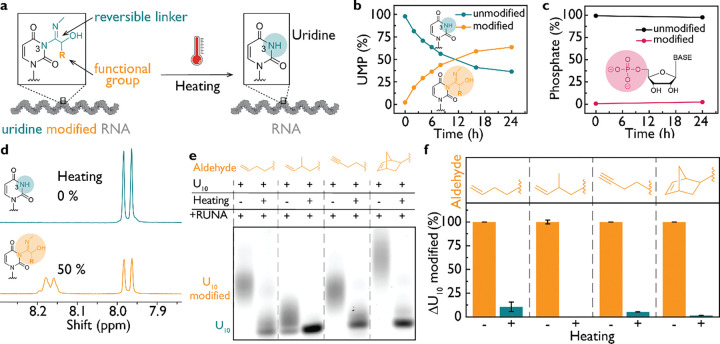
Reversible functionalization of native RNA. (**a**) RNA containing modified uridine is restored to native RNA state by hydrolysis upon heating. (**b**) ^1^H NMR of modified uridine monophosphate (UMP) in the presence of 100 mM MeNC and norbornene aldehyde, pH 8.0, reveals the reaction kinetics, whereas (**c**) ^31^P NMR results reveal that the phosphate group remains unmodified. (**d**) The N3 modification of uridine is removed by heating 95 °C for 15 min at pH 8.0, as demonstrated by ^1^H NMR. (**e**) Using U_10_-Cy3 RNA as a model, we showed that various functional groups, including click chemistry handles such as alkene, alkyne, and strained norbornene are introduced to RNA and removed by heating. (**f**) Gel analysis of normalized modification levels before and after heating shows that most of the modification is removed from RNA.

We next investigated whether the N3 adduct on uridines could be removed controllably to restore native RNA structure (Figure 2a). We first assessed product stability and found that it remained intact for over seven days at room temperature. To test if the adduct could be removed by hydrolysis upon heating, we heated modified UMP and monitored reaction progress by ^1^H and ^31^P NMR spectroscopy at pH 8.0. Upon heating to 95 °C for 15 minutes, the U-N3 adduct was completely converted to native uridine (Figure 2d; Figure S24).

We next evaluated whether this chemistry translated to functionalized oligonucleotides. We labeled rU_10_ by RUNA with 4-pentenal, 2-methyl-4-pentenal, 4-pentynal, or norbornene aldehyde (5-norbornene-2-carboxaldehyde), and upon heating, we observed efficient adduct removal in all constructs (Figure 2e, f; Figure S25). Thus, because the N3 adduct formed by RUNA is thermally labile, any functional group introduced by this method can be efficiently and cleanly reversed to yield the native RNA.

### Selective labeling of intra- and extravesicular RNA by tuning aldehyde permeability

**Figure 3.**
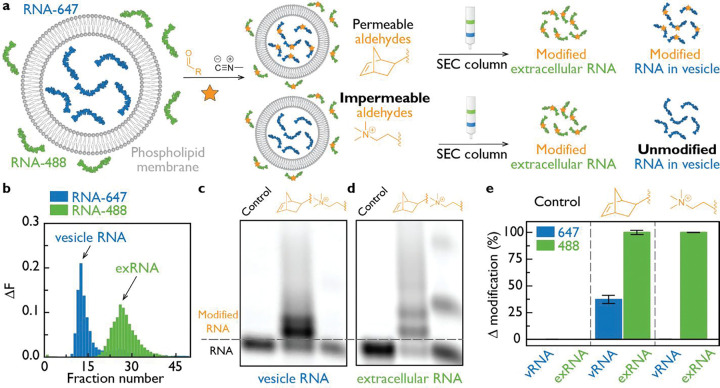
Programmable labeling of extravesicular and intravesicular RNA by modulating aldehyde permeability. **(a)** POPC vesicles encapsulating a 12-nt RNA labeled with ATTO647 (blue; vRNA) were mixed with an identical 12-nt RNA labeled with ATTO488 (green; exRNA) added externally. Aldehyde membrane permeability determines which RNA population is modified. Permeable aldehydes, such as norbornene aldehyde, label both vesicle-encapsulated RNA and extravesicular RNA, whereas the charged, membrane-impermeant betaine aldehyde labels only extravesicular RNA. After the reaction, samples were purified by size-exclusion chromatography (SEC). Fluorescence was recorded for each fraction, and selected fractions were analyzed by denaturing PAGE. **(b)** Representative SEC fluorescence trace. The early eluting peak (ATTO647) corresponds to intact vesicles, while the later peak (ATTO488) represents free extravesicular RNA. **(c, d)** Denaturing gels of the vesicle fraction and extravesicular RNA fraction, respectively. The control sample containing isonitrile but no aldehyde shows no detectable RNA modification. Norbornene aldehyde yields modification of both RNA species, whereas betaine aldehyde modifies only the extravesicular RNA due to its charge-mediated impermeability. **(e)** Quantification of RNA modification levels: no labeling in the control, dual labeling of vRNA and exRNA with norbornene aldehyde, and selective exRNA labeling with betaine aldehyde.

We then tested whether we could distinguish between intra- and extravesicular RNA by tuning the membrane permeability of the aldehyde reagent. We developed a model system in which phospholipid vesicles composed of 1-palmitoyl-2-oleoyl-sn-glycero-3-phosphocholine (POPC) encapsulated an ATTO647-labeled RNA (blue). A second, extravesicular RNA of identical sequence labeled with ATTO488 (green) was added to the bulk solution (Figure 3a, Figure S26).

Membrane-permeable hydrophobic aldehydes can access both RNA pools, unlike charged aldehydes, which can access only extravesicular RNA. To test whether we could asymmetrically label both RNA populations, we used a membrane-permeable norbornene aldehyde and a membrane-impermeant betaine aldehyde. Following RUNA labeling, vesicular RNA (vRNA) and extravesicular RNA (exRNA) were separated by size-exclusion chromatography (Figure S27). Vesicles containing vRNA eluted early, while exRNA eluted later (Figure 3b). Peak fractions corresponding to vRNA and exRNA were collected, and RNA from both fractions was analyzed by denaturing PAGE. Labeling was undetectable when no aldehyde was added (Figure 3c, d). With norbornene aldehyde, both vRNA and exRNA were labeled, consistent with its membrane permeability. In contrast, betaine aldehyde selectively labeled exRNA, reflecting its exclusion from the vesicle interior (Figure 3e). Thus, aldehyde permeability can be leveraged to selectively label extravesicular nucleic acids using RUNA.

### RUNA pinpoints surface DNA as the cue for exosome uptake in macrophages

**Figure 4.**
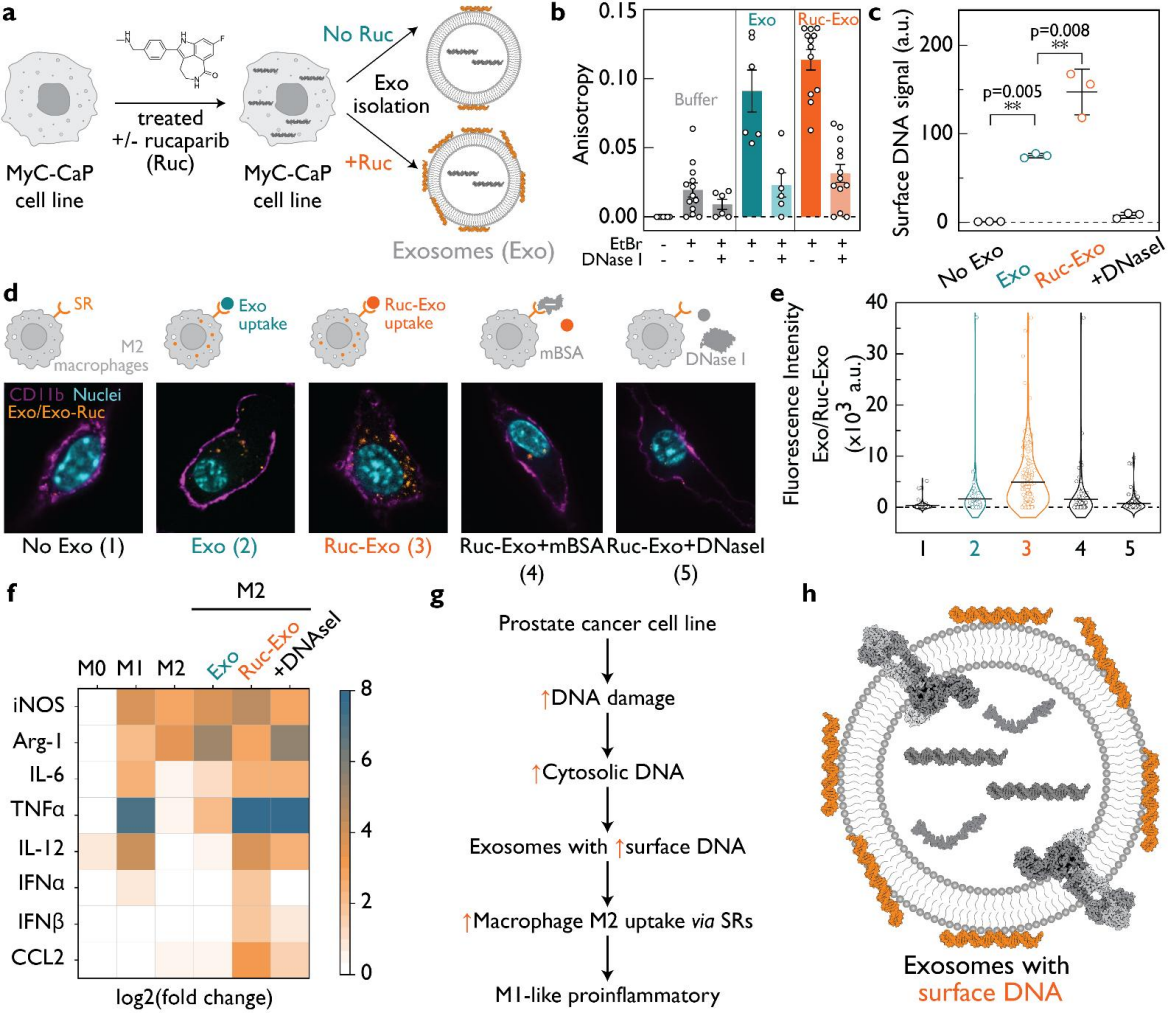
Surface DNA revealed by RUNA enhances macrophage internalization of exosomes. **(a)** Exosomes (Exo) were isolated from MyC-CaP prostate cancer cells treated with or without 0.5 μM rucaparib (Ruc) for 36 h. **(b)** Steady-state fluorescence anisotropy using ethidium bromide revealed a modest increase in signal for exosomes from rucaparib-treated cells (Ruc-Exo). DNase I digestion significantly reduced the anisotropy signal for both Exo and Ruc-Exo samples, indicating the presence of surface-accessible DNA. The sample size was 64. **(c)** RUNA labeling with membrane-impermeant sulfo-Cy3 demonstrated covalent modification of surface DNA. Rucaparib treatment increased Cy3 signal intensity by approximately two-fold, supporting stress-induced elevation of surface DNA abundance. The sample size was 12. **(d)** Fluorescently labeled exosomes with DiO (yellow) were efficiently internalized by M2 macrophages with scavenger receptors (SR) for DNA. (1) M2 macrophages incubated with medium (no Exo) show no DiO signal uptake. (2) Macrophages incubated with Exo show a weak uptake signal. (3) Ruc-Exo show strong uptake and multiple DiO particles. (4) Pretreatment of macrophages with maleylated BSA (mBSA), a scavenger receptor (SR) inhibitor, also limited exosome uptake, implicating surface DNA and scavenger receptors as essential mediators of internalization. (5) Pretreatment of Exo with DNase I eliminated strong DiO signal and abolished uptake. The scale bar is 5 μm. **(e)** The fluorescence intensity of DiO associated with uptake for conditions (1–5) shows statistically significant Exo (p=1.53 × 10^−5^) and Ruc-Exo (p=1.51 × 10^−7^) uptake compared to the no Exo control. Both DNase I and mBSA treatments significantly reduced uptake relative to Ruc-Exo, with Ruc-Exo + DNase I (p = 2.25 × 10^−5^) and Ruc-Exo + mBSA (p = 2.91 × 10^−4^). The sample size was 479 cells for all conditions. Uptake values were log_1+x_-transformed and analyzed by one-way ANOVA with Tukey’s HSD correction. Data are reported as individual points with group means. (**f**) M2 macrophages treated with Exo and Ruc-Exo exhibit a characteristic M1-like proinflammatory cytokine and chemokine profile. (**g**) Proposed model: Genotoxic stress, either intrinsic to prostate cancer cells or enhanced by rucaparib, induces DNA damage, elevating cytosolic DNA levels. This increase is mirrored by higher amounts of DNA on the exosome surface (**h**), enhancing macrophage uptake through SR recognition and potentially triggering immune activation. Together, this model links intracellular DNA damage to extracellular immune signaling *via* DNA-coated exosomes.

We then sought to test whether our differential labeling method could be applied to cell-derived vesicles. Exosomes (Exo) are nanoscale vesicles that cells release into the extracellular environment. They have attracted considerable attention for their burgeoning roles in intercellular communication and in drug delivery. The nucleic acid components of exosomes play key roles in signaling and disease (35). Although transcriptomic and proteomic analyses have catalogued exosomal contents, the precise localization of the nucleic acid components within or on the exosome remains unresolved. To explore the role of DNA associated with exosomes we recreated, *in vitro,* a physiological scenario mimicking a cancer patient undergoing anti-cancer therapy. We cultured c-Myc driven murine prostate cancer cells (MyC-CaP) and treated them with 0.5 μM rucaparib (Ruc), a clinically used PARP inhibitor (36) (Figure 4a). Rucaparib treatment impairs DNA repair, induces genomic stress and promotes the accumulation of cytosolic DNA (37). We sought to test whether these cellular conditions affected the DNA content of secreted exosomes. Exosomes released by MyC-CaP cells with and without rucaparib treatment were isolated and characterized. Rucaparib treatment of MyC-CaP cells did not alter either the abundance or the size distribution of exosomes that they produced (Figure S28–S29). This suggests that exosome biogenesis is largely unaltered by rucaparib. We first probed the DNA content of exosomes by ethidium bromide labeling. If exosomal DNA is present, ethidium bromide intercalates into the DNA, loses its rotational mobility, and its fluorescence anisotropy increases. Exosomes derived from cells with or without rucaparib treatment showed an increase in anisotropy. In contrast, RNase A or proteinase K treatment did not alter the anisotropy, reaffirming that the observed signal was DNA-specific (Figure S30). When exosomes were treated with DNase I, which should selectively degrade exposed DNA, the anisotropy reduced dramatically (Figure 4b), suggesting that much of the exosomal DNA is surface-exposed. Alternatively, the exposed DNA may comprise only a small, but structurally critical fraction, and its removal could expose the major lumenal fraction by disrupting exosome integrity. This could give a false impression that most of the exosomal DNA is surface-exposed.

To distinguish between these two possibilities, we devised a covalent, membrane-delimited labeling strategy based on RUNA to pinpoint the localization of exosomal DNA. We used methyl isonitrile and norbornene aldehyde to conjugate the membrane-impermeant sulfo-Cy3 tetrazine to DNA *via* an IEDDA reaction (Figure 4c, Figure S31). RUNA was applied to equal concentrations of exosomes derived from cancer cells (Exo) and those from rucaparib treated cancer cells (Ruc-Exo), before and after treatment with DNase I. In all cases, free, unreacted dye was removed by stringent ultrafiltration, leaving only covalently attached dye molecules. Because the Cy3 dye is membrane-impermeable, any luminal DNA escapes modification. Thus, any Cy3 signal can be attributed solely to surface-exposed DNA. We found that Exo samples were substantially labeled with Cy3 and that DNase I treatment dramatically reduced the signal indicating that most exosomal DNA is surface-exposed. Surprisingly, this signal nearly doubled in Ruc-Exo samples and was completely lost if samples were pre-treated with DNase I. RUNA reveals that not only is most of exosomal DNA surface-exposed, but its abundance on exosomes nearly doubles in response to the accumulation of DNA damage in cells. We denote this surface-exposed DNA as exosomal DNA hereafter.

Tumors in the murine MyC-CaP model are macrophage-driven. Tumor-associated macrophages (TAMs) account for nearly 50% of the tumor mass, and these TAMs adopt an M2-like anti-inflammatory phenotype that promotes tumor growth. To probe the functional relevance of exosomal DNA derived from cancer cells with and without rucaparib treatment, we tested their effect on M2-polarized macrophages. We labeled Ruc-Exo and Exo samples with Dojindo Exosparkler, a lipophilic fluorescent dye that stains exosome membranes. We then added equal amounts of Exo or Ruc-Exo to cultured M2 macrophages and quantified exosome uptake by fluorescence microscopy (Figure S32; Figure S33–S34). We found that Ruc-Exo was taken up much more efficiently than Exo by M2 macrophages (Figure 4d; Table S2). Scavenger receptors are highly expressed in macrophages and are known to bind and internalize anionic ligands such as duplex DNA (38–42). Experiments in the presence of excess maleylated BSA (mBSA), a well-known competing ligand, revealed that uptake was predominantly *via* scavenger receptors. Furthermore, pre-treating Ruc-Exo with DNase I effectively abolished exosome uptake. Our data establish that exosomal DNA drives exosome uptake by engaging scavenger receptors on M2 macrophages.

To test whether exosome uptake had any functional impact on M2 macrophages, we profiled the cytokines they secreted before and after exosome uptake. We observed that Ruc-Exo uptake led to M2 macrophages showing an increased expression of markers indicative of an activated M1 macrophage cell state. The secretion of type I interferon, IL-12, and TNF-α increased, as did protein expression of iNOS and CCL2. Concomitantly, the levels of proteins such as arginase-1, which is abundant in anti-inflammatory M2 macrophages, decreased. Arginase-1 depletes environmental arginine by hydrolysis into urea and L-ornithine, thereby resolving inflammation. The cytokine/chemokine profile for M2 macrophages treated with Ruc-Exo better resembles that of M1 macrophages, rather than canonical M2 macrophages or those that have taken up exosomes (Exo) from untreated cancer cells. Further, M2-macrophages that take up Ruc-Exo pretreated with DNase I do not express most of these pro-inflammatory markers. Our results reveal that exosomal DNA from drug-treated cancer cells plays a key role in shifting M2-polarized macrophages to a more M1-like state (Figure 4e, Figure S35).

## Discussion

RUNA establishes a new reversible, nucleobase-selective chemistry that unites covalent precision with spatial control. Unlike classical carbodiimide reagents such as EDC or CMC, which irreversibly modify uridines and guanosines and eventually disrupt structure (4), RUNA installs a tunable modification at N3 of U and T in nucleic acids sequence-independently. Unlike acylation-based methods that target only RNA (1), RUNA can be applied to both RNA and DNA. Importantly, one can reverse this modification and recover native RNA and DNA without altering their integrity, a feature that was previously limited to enzymatic or photocleavable systems.

Another distinctive feature is its spatial selectivity, which arises from the modular nature of RUNA. RUNA uses two modules comprising reagents that are simple and commercially available: aldehydes and isonitriles. Whereas most labeling techniques are restricted to either intracellular or free nucleic acids (2), RUNA can selectively address membrane delimited localization by exploiting the membrane permeability of either of its modules. Chemical labeling provides topological specificity, thereby distinguishing between DNA or RNA located inside vesicles and nucleic acids exposed on the outside. Such spatial discrimination has been previously achieved only with sophisticated enzymatic tagging or compartment-specific metabolic labeling (26, 43), both of which require genetic manipulation. RUNA now provides a purely chemical route to access such information.

By applying this chemistry to exosomes, we resolved a previously ill-defined topological feature of DNA in exosomes. The precise localization of DNA in exosomes has limited our mechanistic understanding of exosome function (27, 31). Though prior studies have detected DNA in exosomes (27, 32, 44), it was not known whether it was encapsulated or exposed (27). RUNA provides direct evidence that a significant fraction of exosomal DNA is surface-exposed and that it increases when cells are under genotoxic stress. This observation links intracellular DNA repair dynamics with exosome composition and thus reveals a bridge between genome integrity and exosome-mediated cell-cell communication.

Our findings point to a new role for exosomal DNA. Exosomal DNA is currently considered a passive cargo in exosomes. However, we observe that exosomes leverage this DNA component for cell entry by engaging scavenger receptors. Further, after uptake, changes in the abundance or composition of exosomal DNA, or both, shift M2 macrophages toward an M1-like state. This suggests that tumor-derived exosomes can encode immunomodulatory signals in their nucleic acid content. Our observation may partly explain the immune-modulating effects of PARP inhibitors observed *in vivo* (45), where their activity extends beyond DNA damage repair to include modulation of the tumor microenvironment.

RUNA labeling opens multiple new avenues of investigation. Chemically, it offers a versatile scaffold for temporal and spatial labeling of nucleic acids in living systems, potentially enabling pulse–chase imaging or reversible installation of functional tags. Biologically, it provides a platform to interrogate surface nucleic acids associated with biological membranes such as glycoRNAs, DNA associated with extracellular vesicles, and exosomes (24, 25, 29). This burgeoning class of molecules is being increasingly implicated in cell–cell and immune communication, indicating that chemical accessibility is a key determinant of nucleic acid functionality in biological systems (23, 28, 30). Future applications may include mapping RNA and DNA accessibility across organelle membranes, viruses, or synthetic protocells, and coupling reversible labeling with sequencing to capture transient extracellular states.

## MATERIALS AND METHODS

### RUNA Reaction

RUNA labeling reactions were performed in 20 μL aqueous solutions containing 1 μM Cy3-labeled RNA, 0–200 mM HEPES at the indicated pH, 10–200 mM isonitrile, and 10–200 mM aldehyde. Reactions were incubated at room temperature (18 °C) unless noted otherwise.

### Denaturing Electrophoretic Mobility Shift Assay

Reaction products were analyzed by 20 % (v/v) urea PAGE. Samples were mixed with formamide/EDTA loading buffer, heated, and resolved at constant power. Fluorescent bands were imaged using a Typhoon scanner, and band intensities were quantified using ImageQuant TL.

### ^1^H and ^31^P NMR Analysis

RUNA reactions for NMR were carried out in 500 μL solutions containing 25 mM nucleotide or nucleoside, 200 mM HEPES, 200 mM methyl isonitrile, 200 mM aldehyde, and 10 % D_2_O. Control reactions lacking individual components were prepared in parallel. Spectra were collected on a 400 MHz instrument using water suppression and analyzed in MestReNova.

### Phospholipid Vesicle Preparation for Extravesicular RNA Labeling

Phospholipid vesicles were prepared from POPC by thin-film rehydration in 200 mM HEPES (pH 8.0) containing 3′-ATTO647-labeled 12-nt RNA. Vesicles were sonicated and purified by size-exclusion chromatography on Sepharose 4B. Fractions were monitored by fluorescence to distinguish vesicle-encapsulated RNA from free dye. Vesicle size distributions and encapsulation efficiency were determined by dynamic light scattering.

### Cell Lines and Primary Cells

MyC-CaP prostate cancer cells were cultured in DMEM supplemented with 10 % FBS, 1 % penicillin–streptomycin, 2 % L-glutamine, and 0.2 % Plasmocin. Bone-marrow–derived macrophages (BMDMs) were generated from C57Bl/6J mice and differentiated for 5–7 d in DMEM containing 10 % FBS and 50 ng/mL M-CSF. All cell lines tested negative for mycoplasma.

### Exosome Assays and Rucaparib Treatment

MyC-CaP cells were treated with 500 nM rucaparib for 36 h before exosome collection. BMDMs were polarized to an M2-like state using 20 ng/mL IL-4 for 24 h and incubated with isolated exosomes for cytokine analysis, flow cytometry, or microscopy.

Conditioned media were sequentially centrifuged at 300 × g, 2,000 × g, and 10,000 × g, followed by ultracentrifugation at 100,000 × g for 70 min. Pelleted exosomes were washed once in PBS and quantified by nanoparticle tracking analysis. Size distributions were confirmed by dynamic light scattering.

### RUNA Labeling of Exosome Surface DNA

Exosomes (10^7^ particles) in 20 mM HEPES (pH 7.4) were incubated with 100 mM methyl isonitrile and 100 mM norbornene aldehyde at 4 °C for 12 h. Excess reagents were removed by centrifugal filtration (100 kDa MWCO). Tetrazine–sulfo-Cy3 was added to a final concentration of 5 mM for 2 h, followed by additional filtration. Fluorescence was measured on a Cary Eclipse fluorescence spectrometer (Agilent).

### Exosome Internalization Assays

Equal particle numbers of exosomes from control and rucaparib-treated cells were labeled using the ExoSparkler membrane dye following manufacturer instructions, with an additional ultrafiltration step to remove unbound dye. M2 macrophages were plated on glass-bottom dishes and incubated with labeled exosomes for 40 min. Cells were washed, fixed, and stained with anti-CD11b and Hoechst. Images were collected using a SoRa spinning-disk confocal microscope and analyzed in ImageJ. For inhibition experiments, macrophages were pre-treated with maleylated BSA or exosomes were pre-treated with DNase I.

### Intracellular Staining for Arginase-1 and iNOS / Cytokine, Chemokine Detection from Cell Supernatants

Macrophages were fixed and permeabilized using the BD Cytofix/Cytoperm protocol and stained with PE-conjugated anti-Arginase-1 and APC-conjugated anti-iNOS. Cells were analyzed on an LSR Fortessa cytometer, and data were processed in FlowJo. Cell supernatants were collected 24 h after rucaparib treatment (along with the controls) and used to detect analytes such as cytokines and chemokines using the protocol for LEGENDplex^™^ Mouse Anti-Virus Response Panel (BioLegend).

## Supplementary Material

Supplement 1

## Significance Statement

Pinpointing the localization of RNA and DNA in cells and organelles is central to deriving insights into their biological functions in health and disease. We describe a new method, RUNA, for labeling nucleic acids that is sequence-independent and reversible. By varying RUNA reagents, we can distinguish between nucleic acids that are located either inside or outside of membrane compartments. Using RUNA, we showed that DNA is associated with the exterior surface of exosomes that are secreted by cancer cells. Further, the amount of exosomal DNA increases when the cancer cells are treated with an anti-cancer drug. This surface DNA promotes the uptake of exosomes by innate immune cells known as macrophages and modulates their inflammatory response.
